# Virulome Landscape of Multidrug-Resistant *Escherichia coli* Across Human, Animal, and Environmental Reservoirs

**DOI:** 10.3390/antibiotics15050512

**Published:** 2026-05-19

**Authors:** Eberechi Phoebe Nnah, Arshad Ismail, Akebe Luther King Abia, Sabiha Y. Essack, Daniel Gyamfi Amoako

**Affiliations:** 1 Antimicrobial Research Unit, School of Health Sciences, University of KwaZulu-Natal, Durban 4041, South Africa; abiaakebel@ukzn.ac.za (A.L.K.A.); essacks@ukzn.ac.za (S.Y.E.); amoakodg@gmail.com (D.G.A.); 2 Sequencing Core Facility, National Institute for Communicable Diseases, Johannesburg 2092, South Africa; arshadi@nicd.ac.za; 3 Environmental Research Foundation, Westville 3630, South Africa; 4 School of Pharmacy, University of Jordan, Amman 11942, Jordan; 5 Department of Pathobiology, University of Guelph, Guelph, ON N1G 2W1, Canada

**Keywords:** *Escherichia coli*, virulence genes, One Health, ExPEC, DEC, pathogenicity score, environmental reservoirs

## Abstract

**Background/Objectives:** *Escherichia coli* (*E. coli*) spans commensal, intestinal pathogenic, and extraintestinal pathogenic lineages distributed across human, animal, and environmental reservoirs, yet the extent to which virulence architectures are shared across these compartments remains incompletely understood. Using a One Health framework, we profiled putative virulence determinants in pooled multidrug-resistant (MDR) *E. coli* source groups representing human, animal, and environmental sectors. **Methods:** Virulence genes were predicted with VirulenceFinder, and presence–absence profiles were integrated to define functional composition, sector overlap, source-group distribution breadth, and pathotype-associated signatures. Predicted pathogenic potential was assessed with PathogenFinder and compared with pathogenic family richness. **Results:** Overall, 114 putative virulence genes were detected, with adhesion/colonization functions dominating the virulome (33/114), followed by toxin-associated genes (12/114). A conserved core of 50 virulence genes was shared across all three sectors, including determinants linked to serum resistance (*iss*, *ompT*, *traT*), adhesion (*csgA*, *fimH*), stress adaptation (*terC*), and iron acquisition (*sitA*, *iutA*, *fyuA*). ExPEC-associated determinants were most numerous in environmental source groups (n = 52), whereas diarrheagenic *E. coli* markers were most frequent in animal-associated groups (n = 42). LEE-associated effectors were infrequent and largely absent from human source groups. Despite ecological differences in virulence composition, pathogenicity scores remained consistently high across sectors (0.83–0.92) and showed no significant association with pathogenic family richness (Spearman’s ρ = 0.197, *p* = 0.392). **Conclusions:** Within the limits of pooled source-group analysis, these findings suggest that MDR *E. coli* across One Health compartments shares a broadly distributed, ExPEC-associated virulence repertoire overlaid with sector-specific pathotype signals, underscoring the value of integrated genomic surveillance while highlighting the need for isolate-resolved analysis.

## 1. Introduction

*Escherichia coli* (*E. coli*) is an exceptionally diverse bacterial species that functions as both a commensal and a pathogen. Commensal strains colonize the gastrointestinal tract, supporting digestion and microbial balance. In contrast, pathogenic lineages cause diverse diseases, including diarrhea, urinary tract infections, septicemia, and neonatal meningitis [1]. Virulence factors mediate the ability of *E. coli* to cause disease. They code for adhesins, toxins, invasins, iron acquisition systems, and other factors that help the bacteria colonize, evade the immune system, and damage tissue [2]. These genes, frequently situated on mobile genetic elements such as plasmids, bacteriophages, and pathogenicity islands, form the basis for the classification of *E. coli* into distinct pathotypes, including enteropathogenic (EPEC), enterotoxigenic (ETEC), enterohemorrhagic (EHEC), enteroaggregative (EAEC), and extraintestinal pathogenic *E. coli* (ExPEC) [3].

The locus of enterocyte effacement (LEE) is a widely researched pathogenicity island that encodes the type III secretion system (T3SS) and associated effectors that cause the attaching and effacing (A/E) lesions seen in EPEC and EHEC. These lesions allow for close adhesion to intestinal epithelial cells and disruption of the host cytoskeleton, which causes diarrhea and serious risks such as hemolytic uremic syndrome [4,5]. Key LEE-associated genes include *eae* (encoding intimin) and *tir* (encoding the translocated intimin receptor), which are involved in host cell attachment. The *espA/B/F/J* genes are structural components of the T3SS and effector translocation machinery, whereas non-LEE-encoded effectors (*nleA/B/C*) and *etpD* regulate host signaling and immunological responses. Additionally, *tccP* stimulates actin polymerization and pedestal formation, which improves colonization efficiency [4,5].

The One Health paradigm provides a valuable framework for understanding how human, animal, and environmental health are interconnected, particularly for organisms such as *E. coli*, which circulate across all three compartments [6,7]. Within this continuum, livestock and companion animals can act as reservoirs of pathogenic lineages, while environmental matrices, including surface waters, wastewater, and soils, provide important settings for persistence, amplification, and dissemination [8,9]. Human infection may therefore arise through multiple, overlapping routes, including direct transmission, foodborne exposure, zoonotic contact, and environmentally mediated pathways. Against this background, defining how virulence determinants are distributed across sectors is critical for understanding shared pathogenic potential and for strengthening surveillance strategies within a One Health context.

Importantly, cross-compartment comparisons of virulence gene distribution can distinguish broadly shared from compartment-associated signatures across ecologically linked reservoirs. Such patterns are informative for assessing reservoir connectivity and the broader architecture of pathogen risk, even though they do not in themselves resolve direct transmission pathways or transmission directionality. In this study, the analytical focus was therefore placed on the broader virulence gene repertoire detected across pooled source groups, with pathotype-related interpretation applied subsequently through annotations linked to DEC-, ExPEC-, and other virulence-associated markers. Moreover, despite major advances in genomic approaches, virulence-focused studies of *E. coli* remain disproportionately centered on human clinical isolates, with comparatively limited characterization of animal and environmental reservoirs. This imbalance restricts One Health-level insight into how virulence traits are distributed across ecologically connected compartments [10,11,12]. To address this gap, we characterized the virulence gene repertoire detected across pooled MDR *E. coli* source groups from human, animal, and environmental compartments. Additionally, rather than analyzing predefined pathotypes as separate cohorts, we profiled the broader virulence gene repertoire across pooled multidrug-resistant *E. coli* source groups and subsequently interpreted selected determinants using pathotype-associated annotations, including DEC- and ExPEC-linked markers. Within this framework, LEE-associated genes are considered pathotype-linked virulence signals embedded within the broader virulome, rather than evidence of isolate-level pathotype assignment or pathotype-specific cohort design.

## 2. Results

### 2.1. Distribution of Virulence Factors

**Figure 1:**
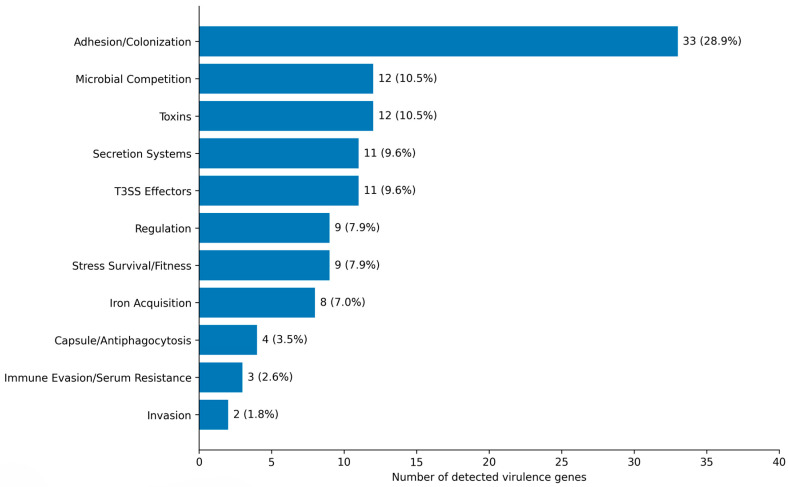
Distribution of virulence genes by function. Horizontal bars show the number and percentage of the 114 detected virulence genes assigned to each functional class. Bars are ordered from most to least abundant functional class.

Virulome analysis identified 114 putative virulence genes, annotated by functional category and pathotype association, across the 21 source groups representing human (n = 2), animal (n = 10), and environmental (n = 9) compartments. Functional classification showed that adhesion- and colonization-associated genes were the most abundant group (n = 33), followed by toxin-associated genes (n = 12) and microbial competition genes (n = 12) (Figure 1). Overall, the detected repertoire was dominated by determinants linked to colonization, persistence, and host interaction rather than by invasion-associated features alone. The full list of detected genes and their functional assignments is provided in Supplementary Tables S1 and S2.

### 2.2. Distribution Across One Health Compartments and Overlap Structure

**Figure 2:**
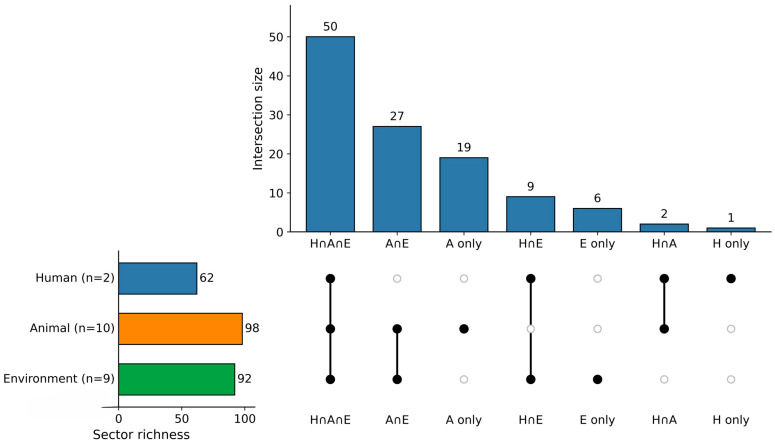
Integrated overlap structure of virulence genes across One Health sectors. The UpSet plot shows sector-level presence/absence intersections for 114 detected virulence genes, including the three-way shared core, pairwise overlaps, and compartment-specific components. The shared core comprised 50 genes detected across human, animal, and environmental sectors, including determinants associated with adhesion/colonization, iron acquisition, serum resistance, and stress adaptation/fitness. H: human; A: animal; E: environment.

Virulence gene richness varied across One Health compartments, with 62 distinct virulence genes detected in human-associated source groups, compared with 98 in animal-associated and 92 in environmental source groups. Despite these differences, the sector-level virulome was anchored by a substantial three-way shared core of 50 genes present across all compartments (Figure 2; Table S3). This shared set included determinants related to adhesion/colonization, iron acquisition, serum resistance, and stress adaptation/fitness. Relative to total richness, the shared core accounted for 80.6% of the human-associated virulome (50/62), compared with 51.0% in animal-associated sources (50/98) and 54.3% in environmental sources (50/92), indicating that human-associated profiles were largely nested within a broader virulence repertoire observed in non-human compartments.

Beyond this common core, additional virulence diversity was concentrated in animal and environmental source groups, which contributed larger exclusive and pairwise-overlap gene sets, whereas human-associated sources contributed very few unique detections (Figure 2). These patterns indicate substantial cross-compartment similarity in virulence profiles at the source-group level, together with a possible asymmetric expansion of accessory virulence content in non-human reservoirs. Given the smaller number of human-associated source groups and the broader heterogeneity of animal and environmental sources, these differences should be interpreted cautiously.

### 2.3. Broadly Distributed Virulence Genes Across Source Groups

**Figure 3:**
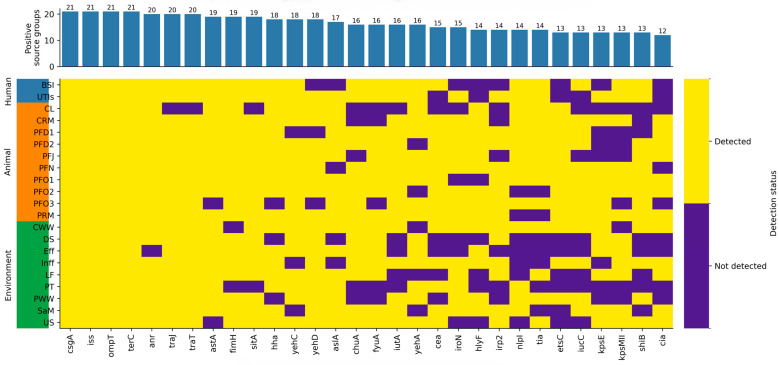
Joint distribution of the 30 most common virulence genes across individual source groups. Columns indicate virulence genes ranked by the number of positive source groups (0–21), not by the number of individual isolates carrying each gene, while rows represent source groups from the human, animal, and environmental sectors. The heatmap displays source-group-level presence/absence patterns and should be interpreted as pooled-source virulence profiles rather than isolate-resolved genotypes. Yellow denotes the presence of a gene, while purple indicates its absence in each source group. Bloodstream infection BSI; urinary tract infection UTI; pig feces December 1 PFD1; pig feces December 2 PFD2; pig feces January PFJ; pig feces November PFN; pig feces October 1 PFO1; pig feces October 2 PFO2; pig feces October 3 PFO3; pig retail meat PRM; chicken litter CL; chicken retail meat CRM; downstream DS; effluent Eff; influent Inf; litter fertilizer LF; pig truck PT; chicken wastewater CWW; pig wastewater PWW; soil after manure application SaM; upstream US.

Comparison across One Health compartments revealed a subset of virulence genes with broad distribution across human, animal, and environmental source groups (Figure 3). Among the 15 most prevalent genes, four (*ompT*, *iss*, *csgA*, and *terC*) were detected in all 21 source groups, while most of the remaining top-ranked genes were present in at least 17 groups spanning all three compartments. These rankings were based on the number of positive source groups in which each gene was detected (0–21) and therefore reflect source-group-level distribution breadth rather than isolate-level prevalence. The most prevalent genes were broadly shared across compartments, whereas lower-frequency genes showed greater variability in their distribution across source groups.

### 2.4. DEC- and ExPEC-Associated Patterns, LEE-Associated Markers, and Core ExPEC Suites Across Compartments

**Figure 4:**
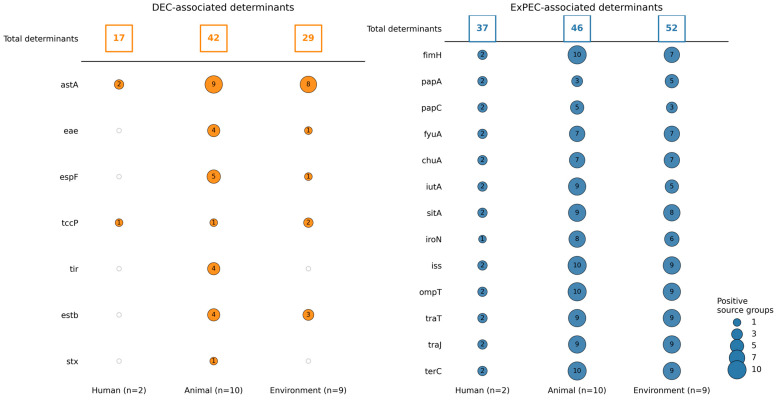
Joint analysis of pathotype-associated virulence determinants and source structure. Two-panel dot plot summarizing DEC- and ExPEC-associated virulence genes across human, animal, and environmental sectors. The top row in each panel shows the total number of associated determinants per sector, and the lower rows show representative hallmark genes, with dot size indicating the number of positive source groups. The figure summarizes sector-level counts and representative markers across compartments. These sector totals reflect source-group-level detection aggregated to the compartment level, based on source-group presence/absence profiles, and should not be interpreted as prevalence estimates.

Cross-compartment comparison of pathotype-associated virulence markers revealed clear sector-level differences between DEC- and ExPEC-linked determinants (Figure 4). DEC-associated markers were most numerous in animal-associated source groups (42), with lower counts in environmental (29) and human-associated groups (17). By contrast, ExPEC-associated determinants were detected across all three compartments, with the highest counts in environmental source groups (52), followed by animal (46) and human-associated groups (37).

In contrast, LEE-associated markers were comparatively rare and unevenly distributed. *eae* was detected in five source groups across animal and environmental compartments but was absent from human-associated sources, whereas *tir* was restricted to four animal-associated groups. Similarly, *espF* was identified only in animal and environmental sources, spanning six groups overall, while *tccP* occurred at low frequency across all three sectors. The canonical ExPEC determinants, however, were consistently detected across human, animal, and environmental reservoirs. These included adhesins (*fimH*, *papA*, *papC*), iron-acquisition systems (*fyuA*, *chuA*, *iutA*, *sitA*, *iroN*), serum-resistance and outer membrane-associated factors (*iss*, *ompT*), plasmid-linked transfer determinants (*traT*, *traJ*), and the fitness-associated gene *terC*. Notably, *iss*, *ompT*, and *terC* were each detected in all source groups, showing the broad distribution of this ExPEC-associated repertoire.

### 2.5. Predicted Pathogenic Potential Across One Health Sectors

**Figure 5:**
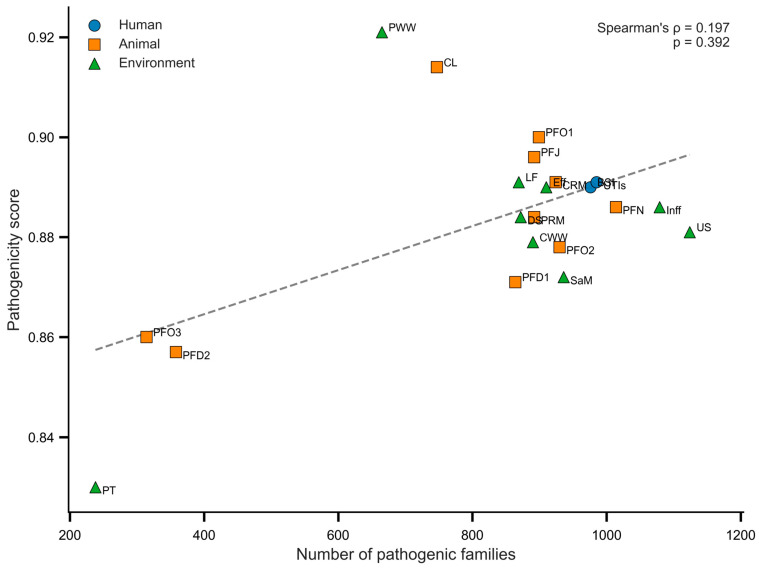
Relationship between pathogenicity score and pathogenic family richness across human, animal, and environmental source groups. Each point represents a source group. Spearman’s correlation showed a weak, non-significant positive association (ρ = 0.197, *p* = 0.392). Bloodstream infection BSI; urinary tract infection UTI; pig feces December 1 PFD1; pig feces December 2 PFD2; pig feces January PFJ; pig feces November PFN; pig feces October 1 PFO1; pig feces October 2 PFO2; pig feces October 3 PFO3; pig retail meat PRM; chicken litter CL; chicken retail meat CRM; downstream DS; effluent Eff; influent Inf; litter fertilizer LF; pig truck PT; chicken wastewater CWW; pig wastewater PWW; soil after manure application SaM; upstream US.

Predicted pathogenicity scores, estimated from assembled source-groups (Methods, Section 4.5), were consistently high across human, animal, and environmental source groups, with only modest variation among sectors (Table S4). Pathogenic family richness, defined here as the number of virulence family classifications represented in the composite pathogenicity analysis, varied across source groups (Figure 5), whereas sector-level pathogenicity means remained comparable, with no significant differences detected among compartments (Kruskal–Wallis, *p* = 0.651) (Tables S4–S6). At the source-group level, animal and environmental groups showed greater dispersion than human-associated groups, which clustered more tightly. This contrast may reflect both biological heterogeneity across source types and uneven source-group representation, particularly the smaller number of human-associated groups.

## 3. Discussion

Using a One Health framework, we mapped virulence genes in drug-resistant *E. coli* across human, animal, and environmental reservoirs by integrating overlap structure, source-group distribution patterns, and pathotype-associated signatures. The pathotype-associated labels were applied after broad virulence screening to aid interpretation of detected determinants, rather than to define predefined analytical cohorts. The resulting virulome showed extensive cross-compartment overlap, with a substantial shared core and only limited compartment-exclusive content, indicating that many virulence-associated traits are distributed across ecologically linked reservoirs rather than confined to a single sector [6,7].

The shared core genes, together with the broad distribution of the most prevalent determinants across source groups, suggest that a large component of the virulome is composed of functions associated with colonization, persistence, host interaction, and ecological fitness. Recurrent detection of adhesion-associated genes such as *csgA* and *fimH*, serum-resistance determinants including *iss*, *ompT,* and *traT*, the fitness-associated gene *terC*, and iron-acquisition systems such as *iutA*, *iroN*, *fyuA*, *chuA*, and *sitA* indicates that these traits are not restricted to human-associated isolates. Similar broad distributions of virulence-linked functions have been reported across human, animal, and environmental *E. coli* backgrounds [7,11,13,14,15], and our data extend these observations by showing source-group-level continuity of these traits within a single regional One Health setting.

At the same time, the data do not support a completely uniform virulence landscape. ExPEC-associated determinants were more numerous in environmental and animal sectors, whereas DEC-associated markers were most concentrated in animal-associated sources. This asymmetry suggests that broadly shared virulence architecture is overlaid by narrower compartment-associated patterns. The environmental enrichment of ExPEC-linked traits and the concentration of DEC-associated markers in animal sources are consistent with reports that wastewater, food animals, and related interfaces can harbor clinically relevant virulence repertoires beyond classical human clinical contexts [3,11,16,17,18,19,20]. However, these contrasts should be interpreted cautiously, as they may reflect not only biological differences across reservoirs but also unequal sampling depth, source heterogeneity, and the pooled source-group design.

LEE-associated markers formed a comparatively limited component of the detected virulome and were largely skewed toward non-human source groups. In particular, *tir* was restricted to animal-associated groups, whereas *eae* and other *esp/nle* markers were not detected in human-associated groups. Given that the sampled human source groups represented bloodstream and urinary tract infection contexts, this pattern is compatible with a predominance of extraintestinal rather than attaching-and-effacing disease biology, although the small number of human-associated source groups limits stronger inference. In contrast to studies in which LEE-associated markers define STEC/EHEC/EPEC-related lineages [3,21], this dataset was characterized more strongly by a broadly distributed ExPEC-associated background than by prominent LEE-associated signatures.

The pathogenicity-score analysis adds a complementary perspective to the gene-distribution results. Although pathogenicity scores remained consistently high across sectors, their weak and non-significant association with pathogenic family richness indicates that inferred pathogenic potential was shaped less by the number of virulence-family classifications than by their composition. Several animal and environmental source groups showed high predicted pathogenicity despite only moderate pathogenic-family richness, suggesting that composite risk may be concentrated in particular virulence configurations rather than scaling directly with family counts. This interpretation is consistent with previous observations that plasmid- and prophage-associated virulence content can amplify pathogenic potential in non-human reservoirs [11].

Several limitations should be considered when interpreting these findings. First, the analyses were conducted at the pooled source-group presence/absence level rather than at the level of isolate prevalence or individual-genome carriage, and sector-level presence was defined by detection in at least one constituent source group. Accordingly, the results describe source-group-level virulence profiles and should not be interpreted as isolate-level prevalence estimates, strain-resolved genotypes, or direct evidence of transmission. Second, the smaller number of human-associated source groups, limited to bloodstream infection and urinary tract infection pools, together with uneven isolate representation across sources, reduced statistical power and comparative resolution for human-sector and source-specific comparisons. This may have constrained detection of human-associated virulence diversity and limited the strength of source-level One Health inference. Third, the greater virulence gene richness observed in animal and environmental compartments may partly reflect deeper sampling, larger numbers of source groups, and greater ecological heterogeneity, rather than intrinsic differences in virulence potential alone. Finally, although all isolates included in this study were multidrug-resistant, the analytical framework was designed to examine virulence gene distribution and predicted pathogenicity rather than resistome composition or resistance–virulence relationships. As a result, the study did not assess co-occurrence, within-genome co-carriage, or potential co-selection between antimicrobial resistance genes, virulence determinants, and mobile genetic elements such as plasmids, integrons, or transposons. Because pooled source-group assemblies were analyzed rather than isolate-resolved genomes, clonal relatedness, resistance–virulence linkage, mobile genetic element context, and transmission directionality could not be resolved. Future studies incorporating balanced isolate-level whole-genome sequencing, integrated resistome–virulome analysis, plasmid reconstruction, and phylogenomics will be required to determine whether multidrug resistance, virulence, and mobile genetic elements are co-selected or co-transmitted across One Health compartments.

However, even within these constraints, the results support a One Health surveillance framework that integrates gene-content profiles with composite pathogenicity measures and genomic context. This combined perspective was more informative than gene counts alone because it highlighted high-scoring non-human source groups that would have been less apparent from richness alone [15,22].

## 4. Materials and Methods

### 4.1. Ethical Consideration

This study is part of a larger program on Antibiotic Resistance and One Health. The Biomedical Research Ethics Committee (reference: BCA444/16) and the Animal Research Ethics Committee (reference: AREC/007/018) at the University of KwaZulu-Natal and the KwaZulu-Natal Provincial Health Ethics Committee (Reference: HRKM108/17KZ_2017RP3_437) provided ethical approval. The Department of Agriculture, Forestry, and Fisheries (DAFF) gave permission to sample animals (Ref 12/11/1/5), while Umgeni Water granted authorization to sample water. The KwaZulu-Natal Department of Health, the uMgungundlovu District Manager, and health facility managers all provided gatekeeper permission.

### 4.2. Study Isolates

This study included 555 multi-drug-resistant (MDR) *E. coli* isolates from earlier studies conducted in the uMgungundlovu District, KwaZulu-Natal Province, South Africa [23,24,25,26,27,28]. The isolates included 12 from humans (isolates implicated in bloodstream and urinary tract infections), 385 from food animals (pigs and poultry samples from farm to table), and 159 from the environment (soil and water bodies). Isolates were preserved at −80 °C at the Antimicrobial Research Unit, University of KwaZulu-Natal. To resuscitate, frozen stocks were thawed and plated onto Eosin Methylene Blue (EMB) agar, which was then incubated at 37 °C for 18–24 h. Distinct *E. coli* colonies were then sub-cultured on nutrient agar to produce pure cultures. Individual colonies were collected with sterile pipette tips and transferred to sterile Eppendorf tubes. To enable comparative analysis across multiple One Health compartments while remaining within sequencing resource constraints, isolates were pooled by source group prior to genomic analysis. Each pooled sample, therefore, represents a composite source group rather than individual isolates. A total of 21 pooled source groups were generated across human, animal, and environmental compartments (Table S7). Pool sizes ranged from 3 to 46 colonies per source group, depending on sample availability. Human-associated pools comprised urinary tract infection (UTI; n = 5) and bloodstream infection (BSI; n = 7) isolates. Animal-associated pools included chicken retail meat (CRM; n = 10), chicken litter (CL; n = 6), pig retail meat (PRM; n = 42), pig feces October 1 (PFO1; n = 40), pig feces October 2 (PFO2; n = 40), pig feces October 3 (PFO3; n = 29), pig feces November (PFN; n = 45), pig feces December 1 (PFD1; n = 44), pig feces December 2 (PFD2; n = 46), and pig feces January (PFJ, n = 42). Environmental pools comprised chicken wastewater (CWW; n = 3), pig wastewater (PWW; n = 38), pig truck (PT; n = 16), upstream (US; n = 36), downstream (DS; n = 10), influent (Inf; n = 25), effluent (Eff; n = 34), soil after manure application (SaM; n = 28), and litter fertilizer (LF; n = 9). Colonies within each source group were combined to form a single composite pool for downstream DNA extraction and sequencing.

This source-group pooling framework was selected as an exploratory design to capture broad virulence gene distribution across One Health compartments rather than to estimate isolate-level prevalence or resolve strain-level relationships. It was intended for distribution-focused virulome mapping rather than balanced isolate-level whole-genome sequencing for prevalence estimation, phylogenomic reconstruction, or isolate-resolved comparative genomics. Pigs are widely known as large reservoirs of virulent *E. coli* strains that can spread to humans via gastrointestinal routes, direct contact, or environmental contamination [29,30,31]. To capture the diversity and evaluate its contribution to pathogenicity, we carried out extensive sampling of pig feces.

### 4.3. Genomic DNA Extraction and Quality Assessment

The GenElute Bacterial Genomic DNA Kit (Sigma-Aldrich, St. Louis, MO, USA) was used to extract genomic DNA from each pooled *E. coli* source group, following the manufacturer’s instructions. DNA concentration and purity were assessed using the NanoDrop™ 1000 spectrophotometer (Thermo Scientific, Wilmington, DE, USA). Colonies were pooled prior to extraction, and where feasible, approximately balanced colony input was used to reduce over-representation of individual colonies within a pool. However, because isolate biomass and DNA yield can vary, perfect isolate-level normalization was not achievable, and downstream analyses were therefore interpreted qualitatively at the source-group presence/absence level rather than as isolate-level quantitative frequencies.

### 4.4. Library Preparation and Sequencing of Pooled E. coli Source Groups

Genomic libraries were prepared from DNA extracted from pooled *E. coli* source groups using the Nextera XT DNA Sample Preparation Kit (Illumina, San Diego, CA, USA), which enables efficient DNA fragmentation and indexing for high-throughput sequencing [32]. Paired-end sequencing was then performed on the Illumina MiSeq platform [33] at the National Institute for Communicable Diseases (NICD) Sequencing Core Facility in Johannesburg, South Africa.

### 4.5. Genomic Data Analysis

Raw sequencing reads generated from pooled *E. coli* source groups were quality-checked using FastQC and trimmed with Trimmomatic by the sequencing facility at the National Institute for Communicable Diseases. Filtered high-quality reads were assembled with SPAdes through the Bacterial and Viral Bioinformatics Resource Center (BV-BRC) assembly pipeline. The resulting assembled contigs from each pooled source-group were used for downstream virulence screening and pathogenicity prediction. Virulence determinants were identified from pooled-source assemblies using VirulenceFinder v2.0 from the Center for Genomic Epidemiology, applying a minimum identity threshold of 90% and a minimum coverage threshold of 60%. Pathogenicity scores were estimated with PathogenFinder [34] using assembled contigs generated for each pooled source-group assembly.

For sector-level analysis, a virulence gene was considered present if it was detected in at least one constituent source group within that sector. Binary presence/absence matrices were used to identify compartment-exclusive genes, pairwise overlaps, and the three-way shared core. Distribution breadth was quantified as the number of positive source groups per gene across all 21 source groups. Unless otherwise specified, gene detections reported in the Results and figure legends refer to source-group-level presence/absence derived from pooled assemblies rather than isolate-level frequency.

Virulence genes annotated as associated with diarrheagenic *Escherichia coli* (DEC) or extraintestinal pathogenic *E. coli* (ExPEC) were further evaluated by integrating pathotype annotations with sector-level presence/absence profiles. Locus of enterocyte effacement (LEE)-associated markers, including *eae*, *tir*, *espA*, *espB*, *espF*, *espJ*, *nleA*, *nleB*, *nleC*, *etpD*, and *tccP*, were specifically screened in the LEE module summary [4]. Overlap structure was visualized using an UpSet plot, broadly distributed genes were summarized in a heatmap, and DEC-/ExPEC-associated determinants were compared across sectors using a dot plot. Data analysis was performed in Python (version 3.11) [35,36,37,38].

### 4.6. Statistical Analysis

Associations between pathogenicity score and pathogenic family richness, defined here as the number of virulence family classifications represented for each source group in the composite pathogenicity analysis, were assessed across the 21 source groups using Spearman’s rank correlation coefficient (ρ) [39]. Sector-level differences in pathogenicity scores among human, animal, and environmental compartments were tested using the Kruskal–Wallis test [40], with Bonferroni-adjusted post hoc pairwise comparisons applied where relevant. All analyses were two-tailed, and statistical significance was set at α = 0.05. The analyses were conducted in Python (version 3.11) using the scientific libraries Pandas, NumPy, SciPy, Seaborn, and Matplotlib. Scatterplots were generated to visualize the relationship between pathogenic family richness and pathogenicity score, with the corresponding test statistics displayed. Virulence gene richness was defined as the number of distinct virulence genes detected within the relevant analytical unit. Accordingly, at the sector level, it represents the number of unique virulence genes identified within a sector based on detection in at least one constituent source group, whereas at the source-group level it denotes the number of distinct virulence genes detected within an individual source group.

## 5. Conclusions

This exploratory study indicates that the virulome of multidrug-resistant *Escherichia coli* is broadly distributed across human, animal, and environmental reservoirs within the sampled One Health framework. At the pooled source-group level, the detected repertoire included a substantial shared core of determinants linked to colonization, persistence, and pathogenic potential. ExPEC-associated determinants were widely represented across compartments, whereas DEC-associated signals were more localized, particularly in animal-associated sources. These patterns suggest that broadly shared virulence architecture coexists with narrower compartment-associated signatures across the human–animal–environment interface. At the same time, these findings should be interpreted as source-group-level distribution patterns rather than as evidence of isolate-level prevalence, direct transmission, or causal reservoir relationships. The consistently high pathogenicity scores observed across sectors further support the view that predicted pathogenic potential is not confined to human-associated sources.

This underscores the value of integrated One Health surveillance for capturing how virulence-associated risk is distributed across linked reservoirs. However, because the present analysis was based on pooled source-group assemblies, it could not resolve strain-level diversity, clonal relatedness, within-genome co-carriage, or resistance–virulence linkage. Future work should therefore prioritize balanced, isolate-resolved genomic approaches that integrate phylogenomics, resistome–virulome profiling, plasmid reconstruction, and mobile genetic element characterization to determine how multidrug resistance and virulence are maintained, associated, and potentially disseminated across interconnected One Health systems.

## Data Availability

All sequencing reads generated from the pooled *E. coli* source groups have been deposited in the NCBI Sequence Read Archive (SRA) under BioProject ID [PRJNA1431768].

## Supplementary Materials

The following supporting information can be downloaded at: https://www.mdpi.com/article/10.3390/antibiotics15050512/s1, Table S1: Functional categories of virulence determinants: definitions, counts, and genes; Table S2: Gene-level mapping to functional categories and associated pathotypes, with aggregated detections by sector; Table S3: 50 common virulence genes across sources; Table S4: Pathogenicity scores, number of pathogenic families, and virulence gene richness across sources and sectors. Table S5: Spearman correlation and Kruskal–Wallis test results for pathogenicity score and pathogenic family richness across One Health sectors. Table S6: Descriptive statistics of pathogenicity scores across One Health sectors. Table S7: Number of composite E. coli colonies per pooled source group.
