# Supplementary material for: Virulome Landscape of Multidrug-Resistant Escherichia coli Across Human, Animal, and Environmental Reservoirs
Source: Antibiotics (Basel). 2026 May 19;15(5):512. doi: 10.3390/antibiotics15050512 (PMC13203363; doi:10.3390/antibiotics15050512)
Supplement: Supplementary file 1 [file antibiotics-15-00512-s001.zip › antibiotics-4229777-supplementary.pdf]

Supplementary Materials

Virulence Gene Distribution in *Escherichia coli* Across Human, Animal, and Environmental Reservoirs: A One Health Analysis

Eberechi Phoebe Nnah[1*], Arshad Ismail[2], Akebe Luther King Abia[1,3], Sabiha Y. Essack[1,4], Daniel Gyamfi Amoako[1,5]

[1] Antimicrobial Research Unit, School of Health Sciences, University of KwaZulu-Natal, Durban 4041,
South Africa; abiaakebel@ukzn.ac.za (A.L.K.A.); essacks@ukzn.ac.za (S.Y.E.); amoakodg@gmail.com (D.G.A.)

[2] Sequencing Core Facility, National Institute for Communicable Diseases, Johannesburg 2092, South Africa; arshadi@nicd.ac.za

[3] Environmental Research Foundation, Westville 3630, South Africa

[4] School of Pharmacy, University of Jordan, Amman 11942, Jordan

[5] Department of Pathobiology, University of Guelph, Guelph, ON N1G 2W1, Canada

* Correspondence: eberechiphoebennah@gmail.com

Table S1: Functional categories of virulence determinants: definitions, counts, and genes

Functional category	Definition	Number of genes	Genes in this category
**Adhesion & colonization**	Adhesins and fimbriae mediating attachment to host tissues and biofilm formation.	33	*afaA, afaB, afaC, afaD, aslA, csgA, eae, faeC, faeD, faeE, faeF, faeH, faeI, faeJ, fdeC, fedE, fimH, focC/sfaE, focG, hra, iha, lpfA, mrkA, nfaE, papA, papC, sfaD, sfaS, yehA, yehB, yehC, yehD, yfcV*
**Capsule & antiphagocytosis**	Capsule biosynthesis and export genes that reduce phagocytosis.	4	*kpsE, kpsM, kpsMII, neuC*
**Immune evasion / serum resistance**	Outer membrane and surface factors that confer resistance to complement-mediated killing.	3	*iss, ompT, traT*
**Invasion**	Factors promoting entry into and traversal of host epithelial or endothelial barriers.	2	*ibeA, tia*
**Iron acquisition**	Siderophore receptors and heme uptake systems enabling iron scavenging in host niches.	8	*chuA, fyuA, ireA, iroN, irp2, iucC, iutA, sitA*
**Microbial competition (bacteriocins/microcins)**	Bacteriocins/microcins that inhibit competing bacteria, promoting niche dominance.	12	*cba, cea, cia, cib, cma, colE9, cvaC, mcbA, mchB, mchC, mchF, mcmA*
**Regulation**	Transcriptional/plasmid regulators controlling expression of virulence determinants.	9	*aalC, aalF, aamR, air, anr, eilA, hha, perA, traJ*
**Secretion system**	Autotransporters and secretion components that export virulence factors to the cell surface or environment.	11	*aaiC, espA, espB, etpD, etsC, pic, sat, sepA, tibC, tsh, vat*
**Stress survival & fitness**	Determinants enhancing survival under acid, heat, oxidative or osmotic stress.	9	*clpK1, clpK2, dhaK, gad, katP, nlpI, shiA, shiB, terC*

**T3SS effectors**	Type III secretion system effector proteins injected into host cells to subvert cellular pathways.	11	*capU, cif, efa1, espF, espJ, espY2, nleA, nleB, nleC, tccP, tir*
**Toxins**	Protein/toxin factors that damage host cells or modulate host responses.	12	*astA, clbB, cnf1, ehxA, esta, estb, hlyA, hlyE, hlyF, senB, stx, usp*

Table S2: Gene-level mapping to functional categories and associated pathotypes, with aggregated detections by sector

Gene	Functional category	Associated pathotypes	Human detections (BSI+UTIs)	Animal detections (CL, CRM, PFD1–PFD2, PFJ, PFN, PFO1–PFO3, PRM)	Environment detections (CWW, DS, Eff, Inff, LF, PT, PWW, SaM, US)
afaA	Adhesion & colonization	UPEC/ ExPEC (ExPEC)	0	0	1
afaB	Adhesion & colonization	UPEC/ ExPEC (ExPEC)	0	0	0
afaC	Adhesion & colonization	UPEC/ ExPEC (ExPEC)	0	0	1
afaD	Adhesion & colonization	UPEC/ ExPEC (ExPEC)	1	1	1
aslA	Adhesion & colonization	UPEC, APEC (ExPEC)	2	8	5
csgA	Adhesion & colonization	UPEC (ExPEC)	2	10	6
eae	Adhesion & colonization	EPEC, EHEC (DEC)	1	3	1
faeC	Adhesion & colonization	ETEC (DEC)	1	2	1
faeD	Adhesion & colonization	ETEC (DEC)	0	1	0
faeE	Adhesion & colonization	ETEC (DEC)	0	1	0
faeF	Adhesion & colonization	ETEC (DEC)	1	1	0
faeH	Adhesion & colonization	ETEC (DEC)	1	0	0
faeI	Adhesion & colonization	ETEC (DEC)	1	2	0
faeJ	Adhesion & colonization	ETEC (DEC)	1	1	0

fdeC	Adhesion & colonization	STEC, UPEC, ExPEC (DEC, ExPEC)	0	0	1
fedE	Adhesion & colonization	ETEC (DEC)	0	1	0
fimH	Adhesion & colonization	UPEC and other EXPEC (ExPEC)	2	10	5
focC/sfaE	Adhesion & colonization	UPEC and other EXPEC (ExPEC)	0	1	2
focG	Adhesion & colonization	UPEC and other EXPEC (ExPEC)	0	0	2
hra	Adhesion & colonization	Several (ETEC, EAEC, UPEC)	1	6	4
iha	Adhesion & colonization	Several (UPEC, EHEC, ExPEC)	1	2	2
lpfA	Adhesion & colonization	EHEC, EPEC (DEC)	1	5	3
mrkA	Adhesion & colonization	UPEC (ExPEC)	0	4	3
nfaE	Adhesion & colonization	EAEC (DEC)	0	0	1
papA	Adhesion & colonization	UPEC and other ExPEC (ExPEC)	2	2	4
papC	Adhesion & colonization	UPEC (ExPEC)	1	5	2
sfaD	Adhesion & colonization	UPEC (ExPEC)	0	1	2
sfaS	Adhesion & colonization	UPEC, NMEC, other EXPEC (ExPEC)	0	1	2

yehA	Adhesion & colonization	Several	2	8	4
yehB	Adhesion & colonization	Several	0	1	2
yehC	Adhesion & colonization	Several	2	9	4
yehD	Adhesion & colonization	Several	2	8	6
yfcV	Adhesion & colonization	UPEC (ExPEC)	1	4	2
kpsE	Capsule & antiphagocytosis	ExPEC, NMEC, UPEC, APEC (ExPEC)	2	7	3
kpsM	Capsule & antiphagocytosis	ExPEC, NMEC, UPEC, APEC (ExPEC)	0	2	1
kpsMII	Capsule & antiphagocytosis	ExPEC, NMEC (ExPEC)	2	6	4
neuC	Capsule & antiphagocytosis	ExPEC, NMEC (ExPEC)	0	7	2
iss	Immune evasion / serum resistance	ExPEC, UPEC, APEC (ExPEC)	2	10	6
ompT	Immune evasion / serum resistance	Several (UPEC, ExPEC, EHEC)	2	10	6
traT	Immune evasion / serum resistance	ExPEC, UPEC, APEC (ExPEC)	2	10	6
ibeA	Invasion	NMEC, SEPEC, APEC, UPEC (ExPEC)	1	2	2
tia	Invasion	ETEC (DEC)	2	6	3
chuA	Iron acquisition	UPEC (ExPEC)	1	9	4

fyuA	Iron acquisition	UPEC and other EXPEC (ExPEC)	1	9	4
ireA	Iron acquisition	UPEC, ExPEC (ExPEC)	2	6	1
iroN	Iron acquisition	UPEC, NMEC, SEPEC APEC (ExPEC)	2	7	5
irp2	Iron acquisition	Several (UPEC, ExPEC, EHEC, AIEC)	1	8	4
iucC	Iron acquisition	APEC, UPEC and other ExPEC (ExPEC)	1	7	3
iutA	Iron acquisition	UPEC and other ExPEC (ExPEC)	2	8	4
sitA	Iron acquisition	ExPEC, APEC, UPEC (ExPEC)	2	10	5
cba	Microbial competition (bacteriocins/microcins)	ExPEC	0	4	0
cea	Microbial competition (bacteriocins/microcins)	APEC (ExPEC)	1	8	4
cia	Microbial competition (bacteriocins/microcins)	Several	1	6	4
cib	Microbial competition (bacteriocins/microcins)	Several	0	2	0
cma	Microbial competition (bacteriocins/microcins)	ExPEC	0	5	2
colE9	Microbial competition (bacteriocins/microcins)	Several	0	0	1
cvaC	Microbial competition (bacteriocins/microcins)	NMEC, SEPEC, APEC, UPEC (ExPEC)	2	6	3

mcbA	Microbial competition (bacteriocins/microcins)	ExPEC, APEC (ExPEC)	0	3	0
mchB	Microbial competition (bacteriocins/microcins)	ExPEC, APEC (ExPEC)	0	4	0
mchC	Microbial competition (bacteriocins/microcins)	ExPEC, APEC (ExPEC)	0	4	0
mchF	Microbial competition (bacteriocins/microcins)	ExPEC	0	7	4
mcmA	Microbial competition (bacteriocins/microcins)	ExPEC	0	4	0
aalC	Regulation	EAEC (DEC)	1	1	0
aalF	Regulation	EAEC (DEC)	0	0	0
aamR	Regulation	EAEC (DEC)	1	1	1
air	Regulation	EAEC (DEC)	1	5	2
anr	Regulation	EAEC (DEC)	2	9	6
eilA	Regulation	EAEC (DEC)	1	7	2
hha	Regulation	Several (EHEC, EPEC, UPEC, ExPEC, APEC)	2	8	5
perA	Regulation	EPEC (DEC)	0	1	0
traJ	Regulation	ExPEC, APEC (ExPEC)	2	10	6
aaiC	Secretion system	EAEC (DEC)	0	2	0
espA	Secretion system	EPEC, EHEC (DEC)	1	4	0
espB	Secretion system	EPEC, EHEC (DEC)	1	3	0
etpD	Secretion system	ETEC, EHEC,	0	3	0

		EPEC (DEC)			
etsC	Secretion system	UPEC, NMEC, APEC, (ExPEC)	1	8	2
pic	Secretion system	Several (EAEC, UPEC)	1	2	1
sat	Secretion system	Several (UPEC, EAEC, DAEC)	1	0	2
sepA	Secretion system	EIEC, EAEC (DEC)	0	3	0
tibC	Secretion system	EAEC, ETEC (DEC)	1	0	1
tsh	Secretion system	APEC, other ExPEC (ExPEC)	0	3	0
vat	Secretion system	APEC, ExPEC (ExPEC)	1	2	1
clpK1	Stress survival & fitness	UPEC (ExPEC)	0	4	1
clpK2	Stress survival & fitness	UPEC (ExPEC)	0	1	2
dhaK	Stress survival & fitness	several	1	3	2
gad	Stress survival & fitness	Several	0	4	2
katP	Stress survival & fitness	EHEC, EPEC (DEC)	0	7	1
nlpI	Stress survival & fitness	EHEC, EPEC, ExPEC	2	6	3
shiA	Stress survival & fitness	EHEC, EIEC (DEC)	2	6	2
shiB	Stress survival & fitness	EHEC, EIEC (DEC)	1	7	3
terC	Stress survival & fitness	Several (ExPEC, EHEC, APEC)	2	10	6
capU	T3SS effectors	EAEC (DEC)	0	1	3

cif	T3SS effectors	EPEC, EHEC (DEC)	1	3	0
efa1	T3SS effectors	EHEC, EPEC (DEC)	1	1	0
espF	T3SS effectors	EPEC, EHEC (DEC)	0	5	1
espJ	T3SS effectors	EPEC, EHEC (DEC)	1	3	0
espY2	T3SS effectors	EHEC & EPEC (DEC)	1	6	2
nleA	T3SS effectors	EPEC, EHEC (DEC)	1	4	0
nleB	T3SS effectors	EPEC, EHEC (DEC)	1	3	1
nleC	T3SS effectors	EPEC, EHEC (DEC)	1	0	1
tccP	T3SS effectors	EHEC, EPEC (DEC)	1	1	2
tir	T3SS effectors	EHEC, EPEC (DEC)	0	4	0
astA	Toxins	ETEC, EAEC, DAEC (DEC)	2	9	5
clbB	Toxins	several	1	0	2
cnf1	Toxins	UPEC, ExPEC (ExPEC)	0	0	2
ehxA	Toxins	EHEC, STEC (DEC)	0	4	0
esta	Toxins	ETEC (DEC)	0	1	0
estb	Toxins	ETEC (DEC	0	4	2
hlyA	Toxins	several (UPEC, ExPEC,	1	1	2

		APEC, EHEC)			
hlyE	Toxins	Several (EHEC, ExPEC)	2	2	2
hlyF	Toxins	APEC, UPEC and other EXPEC (ExPEC)	1	8	3
senB	Toxins	EAEC, EIEC (DEC)	1	1	4
stx	Toxins	STEC, EHEC (DEC)	0	1	0
usp	Toxins	UPEC (ExPEC)	0	3	3

Blood stream infection BSI; urinary tract infectionUTI; pig faeces December 1 PFD1; pig faeces December 2 PFD2; pig faeces January PFJ; pig faeces November PFN; pig faeces October1 PFO1; pig faeces October2 PFO2; pig faeces October3 PFO3; pig retail meat PRM; chicken litter CL; chicken retail meat CRM; downstream DS; effluent Eff; influent Inf; litter fertilizer LF; pig truck PT; chicken wastewater CWW; pig wastewater PWW; soil after manure application SaM; upstream US

Table S3: 50 common virulence genes across sources

Gene	Associated pathotypes
aamR	EAEC (DEC)
afaD	UPEC/ ExPEC (ExPEC)
anr	EAEC (DEC)
aslA	UPEC, APEC (ExPEC)
astA	ETEC, EAEC, DAEC (DEC)
capU	EPEC, EHEC (DEC)
cea	APEC (ExPEC)
chuA	UPEC (ExPEC)
cia	Several
clbB	several
clpK1	UPEC (ExPEC)
cma	ExPEC
cnf1	UPEC, ExPEC (ExPEC)
csgA	UPEC (ExPEC)
cvaC	NMEC, SEPEC, APEC, UPEC (ExPEC)
dhaK	several
espY2	EHEC & EPEC (DEC)
estb	ETEC (DEC)
etsC	UPEC, NMEC, APEC, (ExPEC)
faeC	ETEC (DEC)
fimH	UPEC and other EXPEC (ExPEC)
focC/sfaE	UPEC and other EXPEC (ExPEC)
fyuA	UPEC and other EXPEC (ExPEC)
gad	Several
hha	EHEC, EPEC, UPEC, ExPEC, APEC (several)
hlyA	UPEC, ExPEC, APEC, EHEC (several)
hlyE	EHEC, ExPEC (several)
hlyF	APEC, UPEC and other EXPEC (ExPEC)
hra	ETEC, EAEC, UPEC (several)
ibeA	NMEC, SEPEC, APEC, UPEC (ExPEC)
iha	UPEC, EHEC, ExPEC (several)
ireA	UPEC, ExPEC (ExPEC)
iroN	UPEC, NMEC, SEPEC APEC (ExPEC)
irp2	UPEC, ExPEC, EHEC, AIEC (several)
iss	ExPEC, UPEC, APEC (ExPEC)
iucC	APEC, UPEC and other ExPEC (ExPEC)
iutA	UPEC and other ExPEC (ExPEC)
katP	EHEC, EPEC (DEC)

kpsE	ExPEC, NMEC, UPEC, APEC (ExPEC)
kpsMII	ExPEC, NMEC (ExPEC)
lpfA	EHEC, EPEC (DEC)
mchF	ExPEC
mrkA	UPEC (ExPEC)
neuC	ExPEC, NMEC (ExPEC)
nlpI	EHEC, EPEC, ExPEC
ompT	UPEC, ExPEC, EHEC (several)
papA	UPEC and other ExPEC (ExPEC)
papC	UPEC (ExPEC)
pic	EAEC, UPEC (several)
sat	UPEC, EAEC, DAEC (several)

Table S4: Pathogenicity scores, number of pathogenic families, and virulence gene richness across sources and sectors

S/N	Sector	Sample source	Pathogenicity score	Pathogenic families (n)	Virulence gene richness
**1**	Human	BSI	0.89	976	40
**2**		UTIs	0.891	985	53
		Sector summary	0.891 ± 0.001	Range: 976–985	
**3**	Animal	CL	0.914	747	27
**4**		CRM	0.891	924	43
**5**		PFD1	0.871	864	40
**6**		PFD2	0.857	358	52
**7**		PFJ	0.896	892	39
**8**		PFN	0.886	1014	64
**9**		PFO1	0.9	899	59
**10**		PFO2	0.878	930	48
**11**		PFO3	0.86	314	44
**12**		PRM	0.884	892	48
		Sector summary	0.884 ± 0.018	Range: 358–1014	
**13**	Environment	CWW	0.879	890	52
**14**		DS	0.884	872	33
**15**		Eff	0.89	910	32
**16**		Inff	0.886	1079	41
**17**		LF	0.891	869	43
**18**		PT	0.83	238	24
**19**		PWW	0.921	665	32
**20**		SaM	0.872	936	48

**21**	US	0.881	1124	45
	Sector summary	0.882 ± 0.024	Range: 238–1124	

Abbreviations: Blood stream infection BSI; urinary tract infection UTI; pig faeces December 1 PFD1; pig faeces December 2 PFD2; pig faeces January PFJ; pig faeces November PFN; pig faeces October1 PFO1; pig faeces October2 PFO2; pig faeces October3 PFO3; pig retail meat PRM; chicken litter CL; chicken retail meat CRM; downstream DS; effluent Eff; influent Inf; litter fertilizer LF; pig truck PT; chicken wastewater CWW; pig wastewater PWW; soil after manure application SaM; upstream US. Values are based on source-group level analysis.

Table S5: Spearman correlation and Kruskal–Wallis test results for pathogenicity score and pathogenic family richness across One Health sectors.

Analysis	Variable(s)	N	Correlation/Test Statistic	p-value	Interpretation
**Spearman's rho**	Pathogenicity score vs. Number of pathogenic families	21	$\varrho = 0.197$	0.392	No significant correlation
**Kruskal–Wallis**	Pathogenicity score across sectors (Animal, Environment, Human)	21	$\chi^2 = 0.859$, df = 2	0.651	No significant difference between sectors
**Pairwise comparisons (Bonferroni adjusted)**	Environment vs. Animal	19	Test statistic = 0.622	Adj. p = 1.000	No difference
	Environment vs. Human	11	Test statistic = –4.472	Adj. p = 1.000	No difference
	Animal vs. Human	12	Test statistic = –3.850	Adj. p = 1.000	No difference

Table S6: Descriptive statistics of pathogenicity scores across One Health sectors

Sector	N	Mean Pathogenicity Score	Std. Deviation	Minimum	Maximum
**Animal**	10	0.882	0.020	0.830	0.910
**Environment**	9	0.884	0.018	0.840	0.921
**Human**	2	0.890	0.015	0.875	0.905
**Total**	21	0.883	0.019	0.830	0.921

Table S7: Number of composite *E. coli* colonies per pooled source group

S/N	Sample ID	Sample description	Source	Number of colonies
**1**	UTI	Urinary tract infections	Human	5
**2**	BSI	Blood stream infections	Human	7
**3**	CRM	Chicken retail meat	Animal	10
**4**	CL	Chicken litter	Animal	6
**5**	PRM	Pig retail meat	Animal	42
**6**	PFO1	Pig faeces October 1	Animal	40
**7**	PFO2	Pig faeces October 2	Animal	40
**8**	PFO3	Pig faeces October 3	Animal	29
**9**	PFN	Pig faeces November	Animal	45
**10**	PFD1	Pig faeces December	Animal	44
**11**	PFD2	Pig faeces December	Animal	46
**12**	PFJ	Pig faeces January	Animal	42
**13**	CWW	Chicken wastewater	Environment	3
**14**	PWW	Pig wastewater	Environment	38
**15**	PT	Pig truck	Environment	16
**16**	US	Upstream	Environment	36
**17**	DS	Downstream	Environment	10
**18**	Inf	Influent	Environment	25
**19**	Eff	Effluent	Environment	34
**20**	SaM	Soil after manure	Environment	28
**21**	LF	Litter fertilizer	Environment	9
	**Total**			**555**
